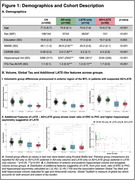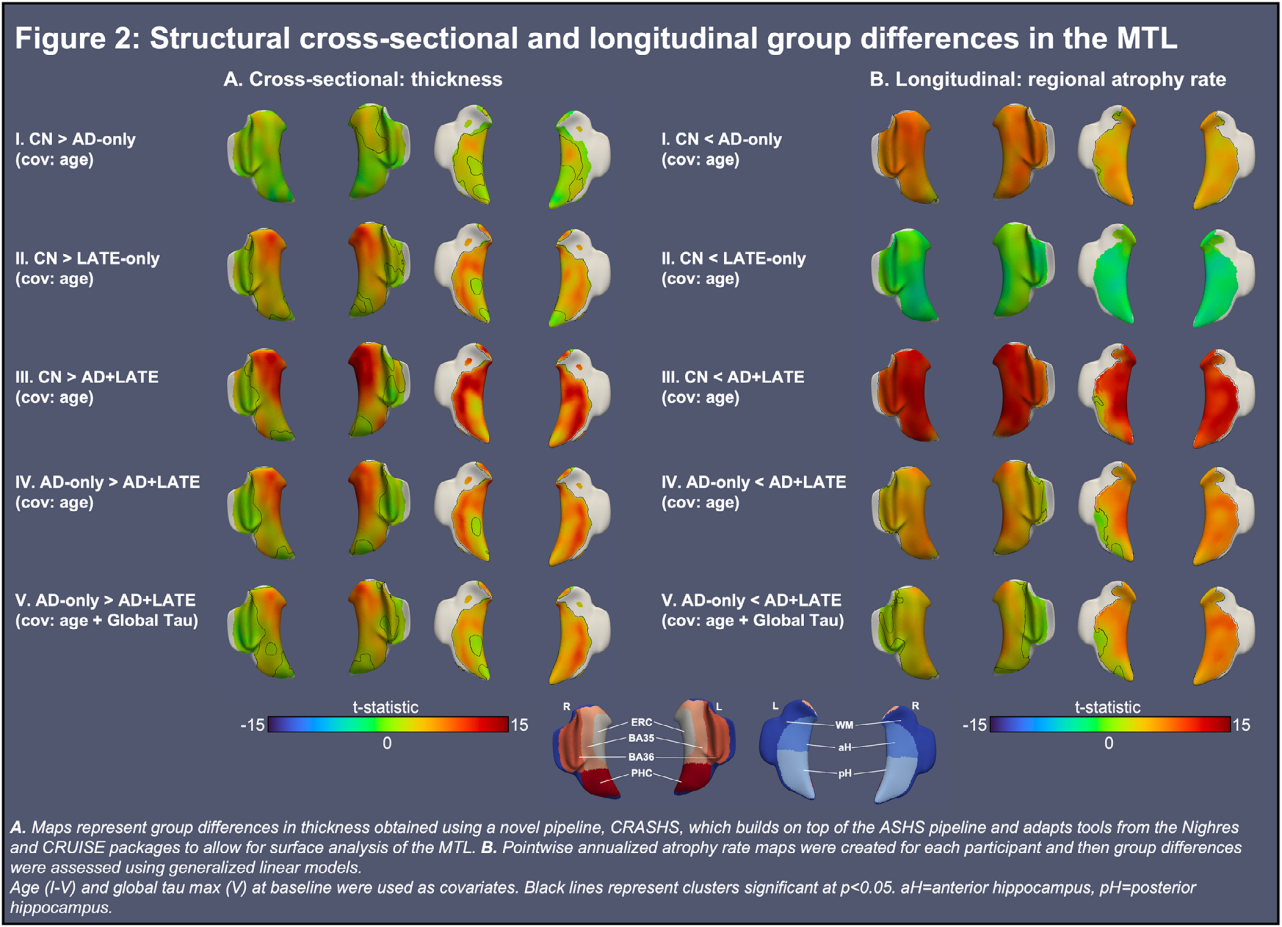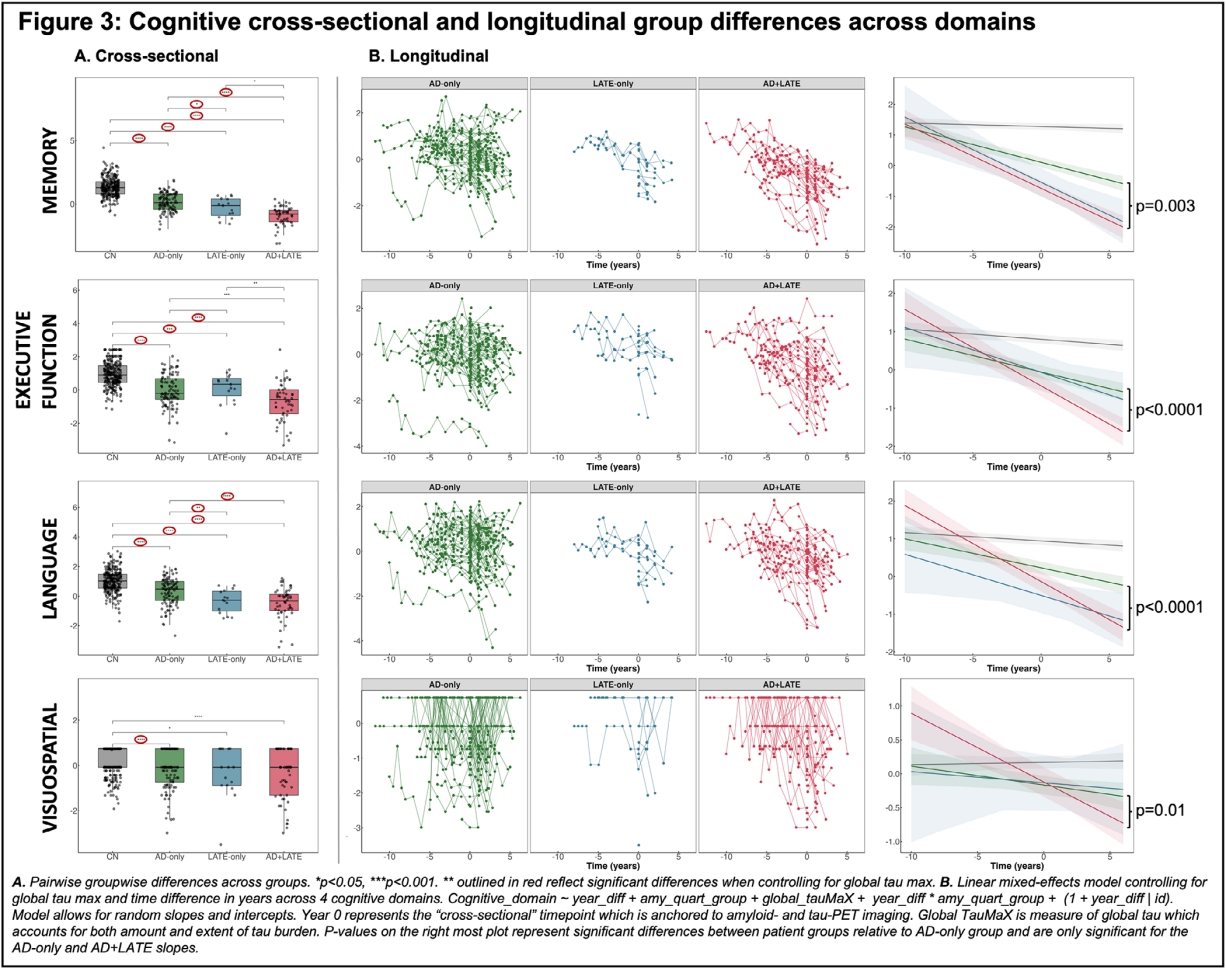# Enrichment of patients with concomitant LATE on the Alzheimer’s disease continuum: comparing structural and cognitive trajectories

**DOI:** 10.1002/alz70862_110000

**Published:** 2025-12-23

**Authors:** Nidhi S. Mundada, Xueying Lyu, Niyousha Sadeghpour, Christopher D. Brown, Emily McGrew, Long Xie, Paul A. Yushkevich, Sandhitsu R. Das, David A. Wolk

**Affiliations:** ^1^ University of Pennsylvania, Philadelphia, PA USA

## Abstract

**Background:**

Overlap in clinical presentations, absence of well‐validated in‐vivo biomarkers for Limbic‐predominant age‐related TDP‐43 encephalopathy (LATE), and frequent co‐occurrence with AD, complicates identifying mixed AD/LATE cases. Autopsy studies suggest greater hippocampal atrophy in AD patients with concomitant LATE. We aimed to identify patients along the AD continuum enriched for LATE by defining the lower quartile of hippocampal volume (HV) as a biomarker for possible LATE and explore atrophy patterns and cognitive profiles.

**Method:**

164 cognitively impaired participants from ADNI with T1‐MRI and amyloid‐ and tau‐PET within 365 days were grouped based on HV quartiles (adjusted for age/intracranial volume) and amyloid status into *suspected* 1) AD‐only (HV>50th‐percentile, amyloid‐positive), 2) LATE‐only (HV<25th‐percentile, amyloid‐negative), 3) AD+LATE (HV<25th‐percentile, amyloid‐positive). We used a novel surface‐based pointwise regional thickness analysis framework to examine cross‐sectional and longitudinal atrophy patterns in the medial temporal lobe (MTL) and determine if AD+LATE showed LATE features beyond hippocampal atrophy. We assessed cross‐sectional and longitudinal differences across 4 cognitive domains (memory, executive function, language, visuospatial).

**Result:**

Suspected‐AD+LATE showed imaging features suggestive of LATE (Figure‐1B(II)), lower MMSE, CDR, and HV, but higher ITG‐tau‐SUVR compared to suspected‐AD‐only (Figure‐1A). Suspected‐AD‐only showed predominant posterior hippocampal atrophy whereas AD+LATE showed more severe anterior hippocampal and amygdala atrophy (Figure‐1B(I)) despite overlapping global tau loads between groups. Patterns of anterior‐posterior atrophy and asymmetry were similar in LATE‐only and AD+LATE (Figure‐1B(II)). Cross‐sectionally, AD+LATE showed significantly lower thickness in MTL‐cortex (Figure‐2A) compared to AD‐only but primarily in anterior MTL‐cortex when controlling for tau. Longitudinally, LATE‐only showed slower atrophy than AD‐only (stronger effects in Figure‐2B(I)>Figure‐2B(II)). AD+LATE showed faster atrophy than AD, however, the effect weakened when controlling for tau (Figure‐2B(IV‐V). Cross‐sectionally, LATE‐only and AD+LATE group showed lower memory and language scores than AD‐only, even after controlling for tau (Figure‐3A); however, longitudinally, AD+LATE declined faster across all domains, differing from AD‐only when controlling for tau, suggesting a more aggressive disease (Figure 3B).

**Conclusion:**

Patients in lower quartile of HV on the AD continuum exhibit LATE‐like patterns, suggesting underlying LATE pathology. A simple HV percentile‐based metric may help identify patients with concomitant LATE and AD with potential relevance for clinical trials and anti‐amyloid therapies.